# The synergistic antitumor effect of Karanahan technology and *in situ* vaccination using anti-OX40 antibodies

**DOI:** 10.32604/or.2025.059411

**Published:** 2025-04-18

**Authors:** VERA RUZANOVA, ANASTASIA PROSKURINA, GENRIKH RITTER, EVGENIYA DOLGOVA, SOFYA OSHIKHMINA, SVETLANA KIRIKOVICH, EVGENIY LEVITES, YAROSLAV EFREMOV, OLEG TARANOV, ALEXANDR OSTANIN, ELENA CHERNYKH, NIKOLAY KOLCHANOV, SERGEY BOGACHEV

**Affiliations:** 1 Laboratory of Induced Cellular Processes, Institute of Cytology and Genetics SB RAS, Novosibirsk, 630090, Russia; 2 Novosibirsk National Research State University, Novosibirsk, 630090, Russia; 3 Center for Shared Use of Microscopic Analysis of Biological Objects SB RAS, Institute of Cytology and Genetics SB RAS, Novosibirsk, 630090, Russia; 4 Department of Microscopic Research, State Research Center of Virology and Biotechnology “Vector”, Koltsovo, 630559, Russia; 5 Laboratory of Cellular Immunotherapy, Research Institute of Fundamental and Clinical Immunology, Novosibirsk, 630099, Russia; 6 Department of Systems Biology, Institute of Cytology and Genetics SB RAS, Novosibirsk, 630090, Russia

**Keywords:** Karanahan technology, OX40, Antitumor immunity, Tumor-initiating stem cells, Systemic inflammatory reaction

## Abstract

**Objectives:**

Currently, there exist two approaches to the treatment of malignant neoplasms: the Karanahan technology and *in situ* vaccination, which are based on chronometric delivery of therapeutic agents to the tumor depending on the characteristics of tumor cells, as well as the immune status. The main purpose of this study was to experimentally prove the feasibility of combining the Karanahan technology and *in situ* vaccination with αOX40 antibodies into a single therapeutic platform to achieve a potent additive antitumor therapeutic effect.

**Methods:**

BALB/c mice grafted with B-cellular lymphoma A20 were treated using the Karanahan technology consisting of intraperitoneal cyclophosphamide administrations and intratumoral DNA injections according to an individually determined therapeutic regimen, together with *in situ* vaccination with αOX40. A pathomorphological analysis of the organs of experimental animals that died during the initial attempt to combine the two technologies was carried out. An analysis of blood cell populations was performed to determine the safe time for antibody administration: the number of immune cells capable of activating systemic inflammation (CD11b+Ly-6C+, CD11b+Ly-6G+, CD3–NKp46+CD11b+), the presence of Fc receptor and OX40 on the surface of these cells, and the number of neutrophils activated to NETosis were analyzed. Based on the analysis results, the antitumor efficacy of various modes of combining the Karanahan technology and *in situ* vaccination was studied.

**Results:**

When αOX40 was administered 5 h after each treatment using the Karanahan technology, mass death of mice caused by systemic inflammation and multiple organ failure was observed. The state of blood cells after the treatment using the Karanahan technology at the time points corresponding to antibody injections was analyzed to elucidate the reasons for this effect. It was found that at some time points, there occurs activation of the immune system and a powerful release (up to 16%) of monocytes and granulocytes carrying Fc receptor and OX40 on their surface into blood; when interacting with αOX40, they can activate the lytic potential of these cells. Activation of neutrophils to NETosis was also observed. Based on these findings, a study was carried out in different time regimes to combine the Karanahan technology and αOX40 injections. When αOX40 was injected into the points of minimal release of myeloid cells into the blood, increased survival rate and the greatest antitumor efficacy were observed: 37% of animals survived without relapses on day 100 after experiment initiation. **Conclusions:** The results obtained indicate that it is possible to combine the Karanahan technology and *in situ* vaccination with αOX40, with obligatory constant monitoring of the number of myeloid cells in peripheral blood to determine the safe time for antibody injection.

## Introduction

### Novel chronometric approaches to cancer therapy

Three new approaches to the treatment of tumors of different etiologies and stages have appeared in modern experimental and clinical practice. These approaches rely on the integral body and tumor response to therapeutic treatment leading to cancer eradication rather than on the “attacking molecule/target” principle.

Those are *in situ* vaccination, metronomic/chronometric chemotherapy with low-dose cyclophosphamide (CP), and the general Karanahan approach, which is based on eradication of cancer stem cells (CSCs). The concepts regarding the fundamental importance of the knowledge about temporal rhythms of the activity of molecular processes taking place in cells, different cellular systems, tissues, organs, tumor cells, and tumor *per se* as a separate aberrant organ (i.e., the main targets of therapeutic drugs), have emerged together with the advent of these technologies [1]. Furthermore, in our opinion, an essential paradigm associated with understanding the body’s individual immune status as a starting point for treating cancer using immunological approaches has appeared [2]. These new concepts have in essence become the ideological foundation of the aforementioned technologies, relying on therapy timing depending on various characteristics of tumor and immune cells and assessment of the system’s immune status.

*In situ vaccination*. The concept of tumor immunogenicity is the ideological foundation of this approach. The global clinical practice demonstrates that oncology patients exhibit different responses to treatment with different immunotherapeutic agents. It turned out that this fact is related to the immunogenicity of a neoplasm in one particular patient: there should exist a neoantigen (neopeptide/neoepitope) that will be recognized by cytotoxic T cells within the MHCII. It was evident that this epitope can appear only as a result of a mutation in the gene encoding tumor protein. The technological capabilities of modern sequencing methods have made it possible to design a panel of genes that most commonly become mutants in certain types of tumors. Therefore, cancer immunotherapy has acquired a set of specific molecules exhibiting a targeted effect in the treatment of an individual tumor [3–6].

On the other hand, a fundamentally different approach was developed in cancer immunotherapy. Rather than being based on systemic administration of a certain drug activating a particular type of immunity, this approach is such an action when the entire immune cell population is simultaneously activated in a small tumor area, and all-encompassing anticancer immunity accompanied by degradation of the primary and distal tumors and cure of the organism. This type of local immunotherapy has been called *in situ* vaccination.

One of the most vivid demonstrations of the potential of using *in situ* vaccination to eradicate experimental tumors is the approach reported in ref. [7], where several immunogenic regrafted and induced experimental tumors were successfully completely cured using the synergistic effect of CG-rich oligodeoxynucleotides (CpG) and anti-OX40 antibodies and the “receptor-ligand platform with simultaneous activation of antigen-presenting cells”.

The core principle of this vaccination involves repolarization and activation of dendritic cells immediately in the tumor nidus by CpG oligonucleotides, where all the possible tumor antigens are present. The primed antigen-presenting cells migrate to lymph nodes, where they meet T cells that have received a signal after the engagement of OX40 receptor and agonistic anti-OX40 antibodies, which enhances the viability of these cells.

*Metronomic/chronometric chemotherapy with low-dose CP*. Much attention has recently been paid to the ability of CP to correct the state of tumor-infiltrating immune cells. Metronomic monochemotherapy with low-dose cyclophosphamide exhibits a stimulatory effect on immune cells, and tumor-infiltrating immune cells such as natural killer cells, NKT cells, and dendritic cells. Repolarization of tumor-associated macrophages is also typical of this therapy. In this regard, metronomic/chronometric chemotherapy with low-dose CP can be characterized as regulatory therapy remodeling the tumor stroma and laying the foundation for the manifestation of antitumor activity by factors of innate and adaptive immunity. Reactivation of the professional properties of immune cells is accompanied by tumor regression [8–10]. This very property of CP used in different anticancer therapies is currently considered therapeutically important. Metronomic chemotherapy with CP combines direct cell-mediated cytotoxicity against cancer cells and degradation of tumor microenvironment [11].

*The Karanahan technology*. The novel Karanahan technology for treating malignancies of different etiology is based on the ability of CSCs to internalize extracellular double-stranded DNA (dsDNA) fragments. The history of developing this technology has been thoroughly described in ref. [12]. The original idea was that extracellular fragments (Tetramethylrhodamine (TAMRA)+ dsDNA probe) delivered into the internal compartments of CSCs may interfere with the chromatin repair process whose damage is caused by cytostatic agents used during chemotherapy. Under certain circumstances, such involvement in repair will have a deleterious effect on CSCs, thus inducing their death. Parameters causing CSC death both under *ex vivo* conditions and directly in the tumor have been identified in multi-year studies. It was elucidated that if the repair cycle of tumor cells is “locked” by triple treatment with moderate-dose CP, accompanied by treatment of the tumor tissue or culture with a therapeutic dsDNA within selected time periods, most tumor cells, including CSCs, will undergo apoptotic degradation. It turned out that a tiny amount of CSCs survive this treatment and continue the cell cycle, which inevitably causes recurrence. We found that after the aforementioned triple treatment, the survived cells synchronously entered the therapy-susceptible cell cycle G1 phase. According to the Karanahan technology schedule, they undergo the final eradication treatment within this time period, and the tumor nidus loses its tumorigenicity. The remnant tumor is lysed by the activated immune system, and animals are either cured from fulminant malignancies or achieve long-term remission. Cell cycle synchronization is related to the sequential repair of CP-induced interstrand crosslinks in euchromatin/ heterochromatin/ centromeric chromatin by the final amount of repair complexes. It is believed that the spatial structure of centromeric heterochromatin is repaired in the last turn because repair sites are inaccessible for repair complexes. In this situation, the pooled repair complexes released after the repair of simple sites simultaneously start repairing interstrand crosslinks of centromeric heterochromatin. Meanwhile, the number of complexes is greater than the number of repair sites. For this reason, both single and multiple damaged sites stochastically present in different cells are repaired simultaneously. Remnant cells synchronously complete the repair and enter the cell cycle, where they undergo final eradication treatment.

The Karanahan technology has been tested in different experimental murine tumor models: Krebs-2 ascites and solid carcinoma, hepatocellular carcinoma (G-29) with ascites, RLS lymphosarcoma, Lewis carcinoma, as well as human tumor models: U87 glioblastoma and primary glioblastoma cultures. Furthermore, Karanahan technology has been tested in 12 pilot clinical trials focusing on breast cancer treatment (for references see [12]).

### The principle of combining the three technologies into a single therapeutic platform

In our theoretical work, we proposed the concept of combining several new techniques based on chronometric drug administration, with each of the techniques having its own therapeutic target and mechanism of action, into one antitumor therapy [13]. We selected the following approaches: the Karanahan technology, metronomic/chronometric chemotherapy with low-dose CP, and *in situ* vaccination. The Karanahan technology was chosen as the basis for the new therapeutic platform.

The Karanahan technology and metronomic/chronometric chemotherapy with low-dose CP have common features, which include the timing and doses of drug administration. In this sense, metronomic/chronometric chemotherapy with low-dose CP is an integral part of the Karanahan technology [14]. The Karanahan technology and *in situ* vaccination also have similar features. Each of these technologies uses DNA-based substances with the same cellular target–dendritic cells. It is worth noting that Karanahan technology also induces total apoptosis of committed cancer cells and launches the molecular events leading to the eradication of CSCs. The use of OX40-specific antibodies as part of *in situ* vaccination leads to activation of the T cell immune response. Thus, the therapeutic effectiveness of Karanahan technology can be further enhanced by activating an adaptive immune response [13].

The total additive therapeutic effect of this exposure is expected to leave no chance for the survival of primary tumors and distant metastases. At the same time, the main postulate of this combined approach is the basic medical principle “do no harm.” We assume that the causal factor for combining the technologies is the timeline factor. It is necessary to exclude the interference of specific mechanisms of action of administered agents by each other. In other words, the combination of approaches requires a preliminary assessment of the time of dendritic cell activation by DNAmix, the time of the innate and adaptive immune system activation by antibodies, the time of CSC eradication, the time of total apoptosis induction, and exclusion of the possibility of their negative effect on each other.

The main goal of this study is to experimentally confirm the feasibility of combining Karanahan technology (metronomic chemotherapy as an integral part) and *in situ*, vaccination using αOX40 into a single therapeutic platform with an additive antitumor therapeutic effect in the treatment of experimental mouse tumors.

## Materials and Methods

### Reagents, equipment, and software

For cell culture, we used: Dulbecco’s Modified Eagle’s Medium (DMEM) (Thermo Fisher Scientific, Cat# 41966029, Waltham, MA, USA), Fetal Bovine Serum (FBS) (Capricorn Scientific, Cat# FBS-11A, Ebsdorfergrund, Germany), penicillin-streptomycin (Biolot, Cat# 1.3.18., St. Petersburg, Russia), β-mercaptoethanol (Merck, Cat# M6250-100 ML, Darmstadt, Germany), mitomycin C (MMC) (Sigma-Aldrich, Cat# M4287-2MG, St. Louis, MO, USA).

For the treatment of experimental mice, we used: Cyclophosphamide (CP) (Baxter Oncology GmbH, Endoxane, Halle, Germany), DNAmix (KARANAHAN LLC), and Anti-OX40 mAb (Becton, Dickinson and Company, Cat# 559861, Franklin Lakes, NJ, USA).

To conduct a pathomorphological analysis, we used neutral paraformaldehyde (Servicebio, Cat# G1101-500 ML, Wuhan, China) and hematoxylin-eosin (Servicebio, Cat#G1076-500 ML, Wuhan, China).

To analyze mouse blood cell populations, we used: Phosphate-buffered saline (PBS) (Servicebio, Cat# G4202-500 mL, Wuhan, China), Fetal Bovine Serum (FBS) (Capricorn Scientific, Cat# FBS-11A, Ebsdorfergrund, Germany), Phorbol 12-Myristate 13-Acetate (PMA) (Sigma-Aldrich, Cat# 19-144, MO, USA), 4′,6-diamidino-2-phenylindole (DAPI) (Sigma-Aldrich, Cat# D9542-1MG, MO, USA), antibodies and isotype controls. The following antibodies were used (BioLegend, San Diego, CA, USA, cat. No. is presented in parentheses): myeloid-derived suppressor cells (MDSCs)—APC anti-mouse/human CD11b Antibody (BioLegend Cat# 101212), PE anti-mouse Ly-6G Antibody (BioLegend Cat# 127607), and FITC anti-mouse Ly-6C Antibody (BioLegend Cat# 128005); natural killer cells—PE anti-mouse CD335 (NKp46) Antibody (BioLegend Cat# 137603), FITC anti-mouse CD3 Antibody (BioLegend Cat# 100204), and APC anti-mouse/human CD11b Antibody (BioLegend Cat# 101212). The following isotype controls were used (BioLegend, cat. No is presented in parentheses): APC Rat IgG2b, κ Isotype Ctrl (BioLegend Cat# 400612), PE Rat IgG2a, κ Isotype Ctrl (BioLegend Cat# 400508), FITC Rat IgG2c, κ Isotype Ctrl (BioLegend Cat# 400705), FITC Rat IgG2b, κ Isotype Ctrl (BioLegend Cat# 400606). Also used polyclonal anti-mouse antibodies to TNFRSF4 (Cloud-Clone Corp, Cat# PAB519Mu01, Hubei, China), Sulfo-Cyanine5 antibody labeling kit (Lumiprobe, Cat# 3321-1rxn, Moscow, Russia) and Fluor® Violet 450 anti-mouse CD16/32 Antibody (Elabscience, Cat# E-AB-F0997Q, Houston, TX, USA).

The following equipment was used in this study: Zeiss Axio Imager M2 (Carl Zeiss Microscopy, Oberkochen, Deutschland), Axio Imager ZI light microscope (Carl Zeiss Microscopy, Oberkochen, Deutschland), BD FACSAria III (Becton, Dickinson and Company, Franklin Lakes, NJ, USA), CO_2_ incubator (Memmert GmbH + Co. KG, Schwabach, Germany) and OLYMPUS IX83P2ZF (Olympus Corporation, Tokyo, Japan).

The following software was used in this study: CASP software Version 2, June 1991 (CASP, Wrocław, Poland), FACSDiVa 8.0.2 software (Becton, Dickinson and Company, Franklin Lakes, NJ, USA), Image J software 1.47v version (Wayne Rasband, Research Services Branch of the National Institute of Mental Health) and Statistica 8 software (StatSoft, Tulsa, OK, USA).

### Study design

At the beginning of the 21st century, two new technologies demonstrating high antitumor efficacy appeared in experimental and clinical practice: the Karanahan approach and *in situ* vaccination with CpG/αOX40 [7,12,15] Tumor immunogenicity is a prerequisite for *in situ* vaccination, while the tumor immune status does not matter in the Karanahan technology. The two technologies have different targets. The concept of this study is that with a proper combination of these technologies, one can ensure that the antitumor effects of one of them will synergistically and additively complement the antitumor effects of the other one. The result will be a new therapeutic regimen combining the benefits of two highly effective antitumor therapies.

This study is an attempt to experimentally find a safe synergistic therapeutic regimen by combining the two technologies for treating malignant A20 B-cellular lymphoma.

The Karanahan technology is chronometric in nature and comprises the administration of a cytostatic agent and DNAmix tied to the repair and cell cycles of a specific tumor. At the first stage of the study, the main parameters of the Karanahan technology for A20 B-cellular lymphoma were determined. Tumor cell culture was exposed to a crosslinking cytostatic agent mitomycin C (MMC) to quantify the duration of the interstrand crosslink repair cycle and identify a time point when either the majority or all tumor cells accumulate in the G2/M phase after long-term arrest due to triple treatment with a cytostatic and a time point when cells synchronously proceed to the G1 phase. Based on the data obtained, a treatment regimen for A20 B-cellular lymphoma using Karanahan technology was compiled.

At the next stage, tumor cells from the culture were transplanted intramuscularly into BALB/c mice to evaluate the therapeutic effectiveness of the Karanahan technology and *in situ* vaccination. The *in situ* vaccination technology with CpG/αOX40 was thoroughly described in ref. [7]. Animals only with developed tumors were included in the experiments. In each experiment, drug injections were started after the tumor size had reached 64 mm^3^. Mice with tumors were randomly divided into groups. An individual mouse was regarded as an experimental unit within the studies. No blinding was used. CP was utilized as a cytostatic agent in mice. The identity of the interstrand crosslinks formation time for MMC *in vitro* and CP *in vivo* had been experimentally demonstrated earlier [12].

In the first step, the effectiveness of tumor treatment using the Karanahan technology (n = 5) and *in situ* vaccination (n = 5) was experimentally compared. Mice with intramuscular grafts treated with saline solution were selected as a control group (n = 5). In the second step, a pilot attempt was made to combine these two technologies. *In situ* vaccination and the Karanahan technology are known to have a similar therapeutic procedure. Both approaches employ DNA-based drugs with the same cellular target: dendritic cells. Therefore, the scheme of CP and DNAmix treatments defined for A20 B-cellular lymphoma using the Karanahan technology was chosen as a basis for combining. Antibodies against the OX40 receptor were injected either together with the DNAmix, or 5 h after DNAmix injections (n = 7 or n = 9, respectively). As a comparison group, mice treated by the Karanahan technology without antibody injections were used (n = 7); mice treated with saline solution were used as a control group (n = 3). When αOX40 had been injected 5 h after administration of DNAmix, rapid death of experimental mice was demonstrated.

At the third stage of the study, numerous analyses were conducted aiming to identify the causes of accidental death of experimental animals. The pilot experiment on combining two modern technologies for tumor therapy was repeated to reveal the causes of mice death. When signs of impending death were noted, mice were sacrificed; their organs and tissues were isolated from experimental and control animals and fixed in 4% neutral paraformaldehyde for pathomorphological analysis. Blood cell populations of mice treated using the Karanahan technology without administration of αOX40 were also analyzed. Two groups of mice with tumors were formed: those treated using the Karanahan technology and a control group of mice treated with a saline solution. The number of immune cells capable of activating systemic inflammation (CD11b+Ly-6C+, CD11b+Ly-6G+, CD3–NKp46+CD11b+), the presence of Fc receptor (FcR) and OX40 on the surface of these cells, and the number of neutrophils activated to NETosis were analyzed.

At the final stage of the study, the therapeutic regimen of antibody administration was adjusted based on the obtained data. Three adjusted experimental regimens were designed to combine the two technologies for treating A20 B-cellular lymphoma (n = 7 or 8). Mice treated using the Karanahan technology without antibody injections were used as a comparison group (n = 5); mice treated with saline solution were used as a control group (n = 5). After the experimental comparison of the three adjusted regimens, the best regimen for combining the two technologies was selected and the feasibility of combining the Karanahan technology with *in situ* vaccination with CpG/αOX40 was experimentally proved.

### Experimental animals

We used female 2- to 6-month-old BALB/c mice (weight, 18–24 g), that were provided by the Common Use Center Vivarium for Conventional Animals of the Institute of Cytology and Genetics of the Siberian Branch of the Russian Academy of Science (Novosibirsk, Russian Federation) as part of a state budget project. Animals were kept in groups of 5–10 mice per cage with free access to food and water. All animal experiments were approved by the Animal Care and Use Committee of the Institute of Cytology and Genetics of the Siberian Branch of the Russian Academy of Science (Protocol No. 8 dated 19 March 2019) and conducted in compliance with the national and international guidelines for the care and humane handling of laboratory animals. The tumor burden did not exceed the recommended dimensions (maximum allowable diameter was 16 mm); animals were sacrificed by cervical dislocation.

### Tumor model

A20 cell line was provided by the Laboratory of Immunogenetics at the Institute of Molecular and Cellular Biology of the Siberian Branch of the Russian Academy of Science (Novosibirsk, Russia). The cell culture was tested for the presence of Mycoplasma. A20 cells were cultured in DMEM supplemented with 10% FBS, 100 U/mL streptomycin, 100 mg/mL penicillin, and 50 μМ β-mercaptoethanol. To produce solid tumors, mice were injected intramuscularly into hind paws, engrafted with 5 × 10^6^ tumor cells diluted in 0.2 mL of physiological saline solution. Tumors were measured with calipers, and tumor volume was calculated as follows: volume = length × width × height.

### Counting TAMRA+ cells in the tumor

All the procedures of TAMRA–DNA probe and incubation with cells were performed as described in ref. [12]. Cell content was analyzed using microscopy. Microscopy was conducted using a Zeiss Axio Imager M2 at the Center for Collective Use of Microscopic Analysis of Biological Objects of the Siberian Branch of the Russian Academy of Science.

### Assessing the DNA repair cycle duration in tumor cells

Tumor cells were incubated with 1 μg/mL MMC in a serum-free medium for 1 h at 37°C in a CO_2_ incubator. The repair cycle was estimated using the comet assay [16] as described in ref. [12]. Comet tail moment was evaluated using the CASP software. Tumor cells before treatment with MMC were used as a control (0 h).

### Assessing cell cycle after triple exposure to the crosslinking agent

The time of terminal Karanahan treatment, leading to complete eradication of CSCs (the time of final eradication treatment) is determined experimentally by daily analysis of the proliferative activity of tumor cells after triple treatment of either mice with a metabolizing crosslinking cytostatic CP or primary tumor cell culture with the crosslinking cytostatic of direct action MMC. There is a time point when either the majority or all cells accumulate in the G2/M phase after long-term arrest due to triple treatment with a cytostatic agent and a time point when cells synchronously proceed to the G1 phase. These parameters are required to calculate the time of administration of a cytostatic agent and the DNAmix. In our earlier article, it was shown that CSCs do not internalize the dsDNA probe in the G2/M phase of the cell cycle [12]. The cytostatic agent (or its metabolite), in turn, is delivered into the cell at any phase of the cell cycle, including the G2/M phase. However, cytostatic-induced interstrand crosslinks can be detected only in the G1 and then the S phases by the presence of functional double-strand breaks [12].

For this reason, when determining the time regimen for the Karanahan technology, the final cytostatic injection is made at the time point when cells have already entered and remain in the G2/M phase. DNAmix is administered at the next time point when cells have entered the G1 phase. The time of maximum accumulation of double-strand breaks, determined in the first part of the repair cycle analysis, is taken into account. This time instant often coincides with the time point at which cells have entered the G1 phase, however, it is paramount in determining the timing of DNAmix administration. Since the G2/M transition lasts approximately 4 h, and the time of maximum accumulation of double-strand breaks always exceeds 6 h, the time of DNAmix administration always coincides with the G1 phase, which is sensitive to internalization.

A20 B-cellular lymphoma cells were exposed thrice to 1 μg/mL MMC with a 54-h interval *in vitro*. On days 4–9 after the first treatment with the crosslinking cytostatic, tumor cells were sampled for cell cycle profiling with propidium iodide, which was carried out as described in ref. [12] using a BD FACSAria III cell sorter and the FACSDiVa software at the Center for Collective Use of Flow Cytofluorometry of the Institute of Cytology and Genetics of the Siberian Branch of the Russian Academy of Science. Tumor cells before treatment with MMC were used as a control (day 0).

### Treatment of A20 B-cellular lymphoma

*In situ* vaccination involves intratumoral administration of immunostimulatory agents, which induces a local T-cell immune response that amplifies and attacks tumors in the whole body. In 2018, Sagiv-Barfi et al. demonstrated the high efficiency of one of the *in situ* vaccination options: a combination of an unmethylated CG-rich oligodeoxynucleotide (CpG), a Toll-like receptor 9 ligand, and αOX40 [7]. CpG introduced into the tumor was shown to activate antigen-presenting dendritic cells and simultaneously induce OX40 expression on the surface of CD4+ T-cells in the tumor microenvironment. OX40 is a co-stimulatory molecule belonging to the tumor necrosis factor receptor superfamily (TNFRSF); it is expressed on both activated effector T-cells and regulatory T-cells. Signaling through OX40 promotes activation of effector T-cells and inhibits the function of regulatory T-cells. The addition of an agonistic αOX40 provides a synergistic stimulus that induces an antitumor immune response, resulting in resorption of distant regions of developed tumors. The principle of immune response induction and amplification by this maneuver is as follows. Tumor antigens, which are always present at the tumor site due to the continuous death of tumor cells, are presented by CpG-activated dendritic cells. Meanwhile, OX40 expression is induced in effector T-cells. Injection of an agonistic αOX40 activates these cells and triggers their interaction with the antigen presented by activated dendritic cells, thus eliciting an immune response.

**Figure 1:**
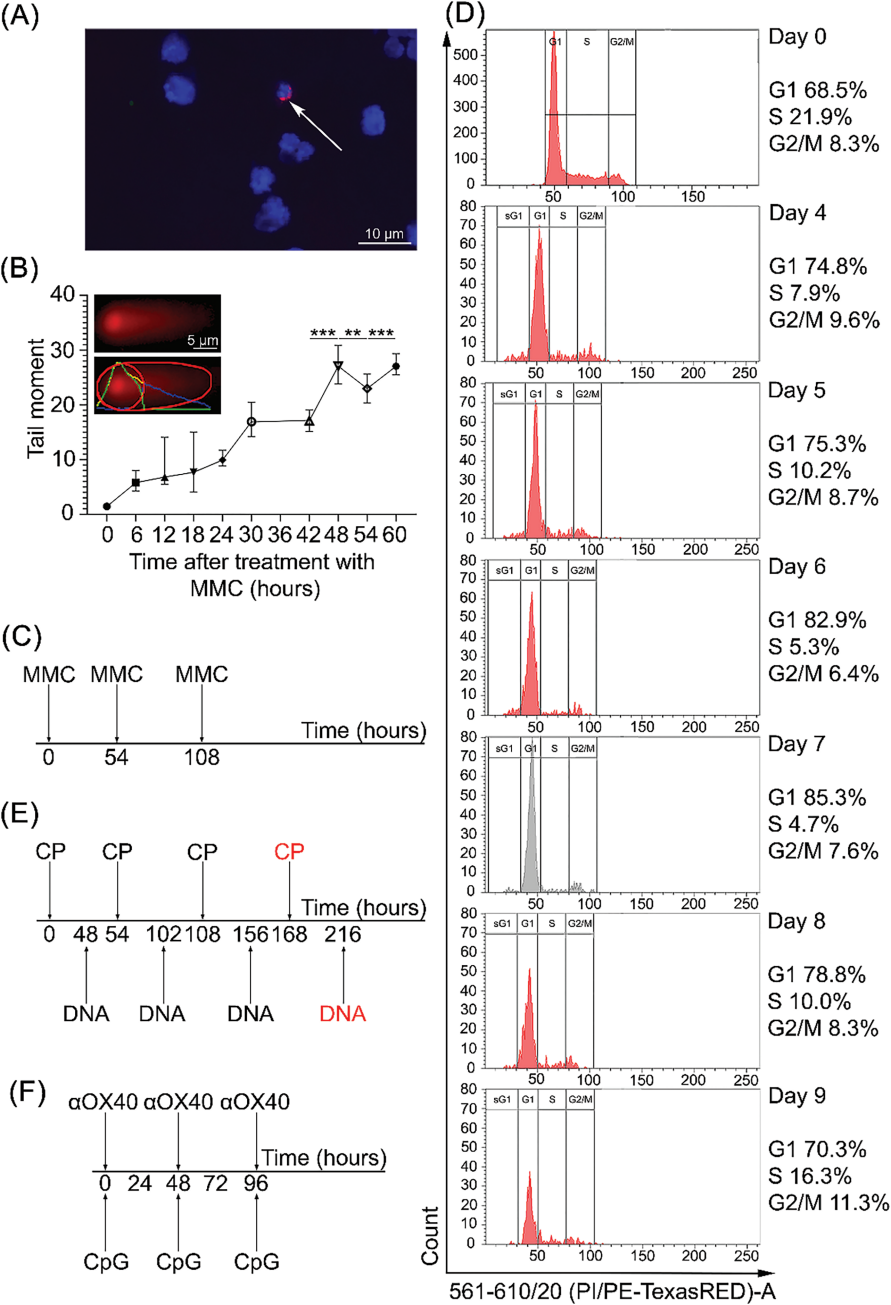
Determination of the basic time parameters of the Karanahan technology for treating A20 B-cellular lymphoma [15]. **(A)** Internalization of Tetramethylrodamine (TAMRA)-labeled DNA probe in tumor cells. The cells were coated with a drop of Antifade 1,4-diazabicyclo [2.2.2]octane (DABCO) with the addition of 0.5 µg/mL of 4′,6-diamidino-2-phenylindole (DAPI). The arrow indicates a specific TAMRA signal. **(B)** Cell repair cycle analysis. Changes in the number of double-strand breaks in tumor cells after treatment with the crosslinking cytostatic agent mitomycin C (MMC) were studied by the comet assay. The figure shows representative images of the comet. ** – differences between points are significant with *p*-value < 0.01; *** – differences between points are significant with *p*-value < 0.001; Mann–Whitney U test. **(C)** Therapeutic scheme of triple treatment of cells with a cytostatic agent MMC developed based on repair cycle parameters. **(D)** Determination of the day for tumor cell synchronization after triple treatment *in vitro* with 1 μg/mL MMC. Cell cycle analysis was performed using cell staining with propidium iodide. The *X*-axis represents the relative DNA level determined based on propidium iodide fluorescence; the *Y*-axis represents the number of cells with the corresponding DNA level. The percentage of cells at corresponding cell cycle phases is presented next to the graphs. The graph demonstrating the moment of synchronization in G2/M is shown in gray. Day 0–A20 B-cellular lymphoma cells before treatment. **(E)** Treatment regimen using the Karanahan technology for A20 B-cellular lymphoma in BALB/c mice, including injections of 100 mg/kg cyclophosphamide (CP) intraperitoneally and 0.5 mg of composite double-stranded DNA preparation (DNAmix) (in the Fig. this is indicated as DNA) intratumorally per mouse. **(F)** *In situ* vaccination regimen for A20 B-cellular lymphoma in BALB/c mice, including injections of 4 μg of αOX40 and 50 μg of CG-rich oligodeoxynucleotide (CpG) intratumorally [7].

Sagiv-Barfi et al. used the following treatment regimen in their study: a combination of αOX40 (4 μg) and CpG oligodeoxynucleotide (50 μg) was injected into one tumor site (locally treated site) three times with a 48-h interval (Fig. 1F). It is the regimen we reproduced completely in our study.

The experiments were carried out at the Institute of Cytology and Genetics of the Siberian Branch of the Russian Academy of Sciences (Novosibirsk). A total of 5 × 10^6^ A20 B-cellular lymphoma cells were grafted intramuscularly in both femoral regions of mice. When the tumor size reached 64 mm^3^, mice were injected with the agents.

The “Karanahan” group received 100 mg/kg of CP intraperitoneally and 0.5 mg/mouse of DNAmix intratumorally, in accordance with the selected therapeutic regimen for A20 B-cellular lymphoma (standard treatment, Fig. 1E). DNAmix is the subject of industrial property of KARANAHAN LLC [17]. The “αOX40+CpG” group received 50 μg of CpG (~2.5 mg/kg) and 4 μg of Anti-OX40 mAb (~0.2 mg/kg) intratumorally thrice with a 48-h interval (Fig. 1F) according to the published therapeutic regimen for *in situ* vaccination [7]. The group of tumor-bearing mice (control group) received similar injections of saline. Intratumoral injections were made only into the right paw (treated tumor). During the experiment, tumor size, relapse development time, and mouse survival time were measured.

### Pilot study on the combination of the Karanahan technology and in situ vaccination with CpG/αOX40

A total of 5 × 10^6^ A20 B-cellular lymphoma cells were grafted intramuscularly in both femoral regions of mice. When the tumor size reached 64 mm^3^, mice were injected with the agents. The “Karanahan” group received standard treatment (Fig. 1E). “Karanahan+αOX40” groups received standard Karanahan treatment (Fig. 1E) with additional injections of 8 μg αOX40 intratumorally immediately or 5 h after each DNAmix injection. The group of tumor-bearing mice (control group) received similar injections of saline. Intratumoral injections were made only in the right paw (treated tumor). During the experiment, tumor size, relapse development time, and mouse survival time were measured.

### Pathomorphological analysis of mouse organs

The pilot experiment on combining the Karanahan technology and *in situ* vaccination with CpG/αOX40 was repeated. The “Karanahan+αOX40” group received standard Karanahan treatment (Fig. 1E) with additional injections of 8 μg αOX40 intratumorally 5 h after each DNAmix injection. The group of tumor-bearing mice (control group) received similar injections of saline. Intratumoral injections were made only into the right paw (treated tumor). When signs of impending death were noted (hump formation, sudden weight loss, and decreased locomotor activity), mice were sacrificed by cervical dislocation. Organs (liver, spleen, small and large intestines, and lungs) and treated tumors were isolated from experimental and control animals (tumor-bearing mice) and fixed in 4% neutral paraformaldehyde. Organ samples were dehydrated in increasing ethanol concentrations, cleared in xylene, and embedded in paraffin. Paraffin sections up to 5 µm thick were stained with hematoxylin and eosin. Visualization of preparations and microphotography were conducted using an Axio Imager ZI light microscope.

### Isolation of blood mononuclear cells

Blood was isolated during mouse decapitation after cervical dislocation, EDTA was immediately added to the final concentration of 7 mM, and erythrocytes were lysed in a buffer containing 130 mM ammonium chloride. Mononuclear fraction cells were resuspended in PBS, and cell concentration was calculated using the Goryaev chamber.

### Аnalysis of changes in the number of myeloid-derived suppressor cells and natural killer cells among the blood mononuclear of mice treated using the Karanahan technology

A total of 5 × 10^6^ A20 B-cellular lymphoma cells were grafted intramuscularly in the right femoral region of mice. When the tumor size reached 64 mm^3^, mice were injected with the agents. The “Karanahan” group received standard treatment (Fig. 1E). The group of tumor-bearing mice (control group) received similar injections of saline. To assess changes in the number of MDSCs and natural killer cell populations, blood mononuclears were isolated 5 h after each Karanahan treatment (one treatment = CP + DNAmix). Meanwhile, blood mononuclear cells were isolated from tumor-bearing (control) and intact mice. PBS containing 10% FBS was added to all cells followed by cell incubation for 10 min at room temperature to block non-specific binding. The studied cells (2 × 10^5^) were incubated with 0.25 µg of antibodies and isotype controls at room temperature in the dark for 30–60 min. Flow cytometry analysis was carried out on a BD FACSAria III cell sorter at the Center for Collective Use of Flow Cytofluorometry of the Institute of Cytology and Genetics of the Siberian Branch of the Russian Academy of Science.

### Assessment of the number of MDSCs with the CD11b+Ly-6G+ phenotype activated to NETosis after using the Karanahan technology

A total of 5 × 10^6^ A20 B-cellular lymphoma cells were grafted intramuscularly in the right femoral region of mice. When the tumor size reached 64 mm^3^, mice were injected with the agents. The “Karanahan” group received standard treatment (Fig. 1E). The group of tumor-bearing mice (control group) received similar injections of saline. To assess the number of cells activated to NETosis, blood mononuclears were isolated 5 h after the first and fourth Karanahan treatments. At the same time, blood mononuclear cells were isolated from tumor-bearing mice (control mice). The studied cells (2.5 × 10^5^) were incubated with 0.5 μg of antibodies in a 24-well plate (1.864 cm^2^ growth area) in 500 μL of DMEM per well. The following antibodies were used: MDSCs (granulocytes)—APC anti-mouse/human CD11b Antibody, PE anti-mouse Ly-6G Antibody. To induce NETosis, PMA was added to each well to a final concentration of 32 nM. Blood mononuclear cells were incubated with antibodies and PMA for 3 h at 37°C in a CO_2_ incubator. DAPI was then carefully added to each well, preventing resuspension and mixing of the cells, to a final concentration of 1.44 μM. Blood mononuclear cells were incubated with DAPI for 15 min at 37°C in a CO_2_ incubator. The number of cells activated to NETosis was assessed using confocal microscopy on an OLYMPUS IX83P2ZF at the Center for Collective Use of Microscopic Analysis of Biological Objects of the Siberian Branch of the Russian Academy of Sciences. The percentage of CD11b+DAPI+Ly-6G+ cells among all CD11b+ cells was estimated using the Image J software.

### Labeling of αOX40

Polyclonal anti-mouse antibodies to TNFRSF4 were labeled using the Sulfo-Cyanine5 antibody labeling kit according to the manufacturer’s instructions.

### Assessment of the number of MDSCs carrying FcR and OX40 on their surface after using the Karanahan technology

A total of 5 × 10^6^ A20 B-cellular lymphoma cells were grafted intramuscularly in the right femoral region of mice. When the tumor size reached 1.4 сm^3^, mice were injected with the agents. The “Karanahan” group received standard treatment (Fig. 1E). The group of tumor-bearing mice (control group) received similar injections of saline. To assess the number of MDSCs with the CD11b+Ly-6G+/CD11b+Ly-6C+ phenotype carrying FcR and OX40 on their surface, blood mononuclear cells were isolated on day 6 after the initiation of the Karanahan therapy. To block non-specific binding, PBS containing 10% FBS was added to all the cells, followed by cell incubation for 10 min at room temperature. The studied cells (2 × 10^5^) were incubated with 0.25 µg of antibodies at room temperature in the dark for 30–60 min. The following antibodies to MDSCs were used: MDSCs—APC anti-mouse/human CD11b Antibody, PE anti-mouse Ly-6G Antibody, and FITC anti-mouse Ly-6C Antibody. The following antibodies were used: anti-FcR antibody–Fluor® Violet 450 anti-mouse CD16/32 Antibody, anti-OX40 antibody–Sulfo-Cyanine5 fluorophore-labeled antibody. Flow cytometry analysis was carried out on a BD FACSAria III cell sorter at the Center for Collective Use of Flow Cytofluorometry of the Institute of Cytology and Genetics of the Siberian Branch of the Russian Academy of Science.

### Combined therapy using the Karanahan technology and in situ vaccination with CpG/αOX40

A total of 5 × 10^6^ A20 B-cellular lymphoma cells were grafted intramuscularly in the right femoral region of mice. When the tumor size reached 64 mm^3^, mice were injected with the agents. The “Karanahan” group received standard treatment (Fig. 1E). The “Karanahan+αOX40 (53, 221)” group received standard Karanahan treatment (Fig. 1E) with additional injections of 8 μg αOX40 intratumorally 5 h after the first (53 h) and fourth (221 h) DNAmix injections. The “Karanahan+αOX40 (167, 335)” group received standard Karanahan treatment (Fig. 1E) with additional injections of 8 μg αOX40 intratumorally 5 h after the third (167 h) DNAmix injection and 335 h after the first injection of CP. The “Karanahan+αOX40 (107, 167, 335)” group received standard Karanahan treatment (Fig. 1E) with additional injections of 8 μg αOX40 intratumorally 5 h after the second (107 h) and third (167 h) DNAmix injections and 335 h after the first injection of CP. The group of tumor-bearing mice (control group) received similar injections of saline. During the experiment, tumor size, relapse development time, and mouse survival time were measured.

### Statistical analysis

Statistical analysis was performed using the Statistica 8 software. The validity of differences was evaluated using the Mann–Whitney U test or analysis of four-field contingency tables. The confidence level (*p*-value) is indicated in the figure caption for each experiment.

## Results

### Determination of the main parameters of the Karanahan technology in the mouse A20 B-cellular lymphoma model

*Internalization of TAMRA-labeled DNA probe by A20 B-cellular lymphoma cells*. The main target of new antitumor therapy is CSCs capable of internalizing dsDNA. In this regard, at the first study stage, it was necessary to determine the presence of target CSCs in the A20 B-cellular lymphoma culture, based on internalization of the extracellular DNA probe labeled with the fluorescent dye TAMRA [12].

It was shown that the A20 B-cellular lymphoma contains 0.4% TAMRA+ CSCs (Fig. 1A).

*Analysis of the time parameters of the repair cycle of A20 B-cellular lymphoma cells after treatment with MMC using the comet assay*. The general ideology of assessing the duration of the repair cycle of interstrand crosslinks induced by a cytostatic agent was described in the [12] and is associated with determining the time of formation and maximum accumulation of double-strand breaks (the phase of nucleotide excision repair) and the recovery time of double-strand breaks (the phase of homologous recombination).

It was found that in A20 B-cellular lymphoma cells the duration of the interstrand crosslink repair cycle after treatment with a cytostatic agent is 54 h. The maximum number of double-strand breaks is observed 48 h after the treatment with a cytostatic agent (Fig. 1B). This time point is used to administer DNAmix when plotting the time schedule for Karanahan technology.

Based on the analysis results, a scheme for three treatments of A20 B-cellular lymphoma cells with a crosslinking cytostatic was obtained (Fig. 1C).

*Determination of the time of A20 B-cellular lymphoma cells synchronization*. To analyze the synchronization time, A20 B-cellular lymphoma cells were treated *in vitro* with the crosslinking cytostatic of direct action MMC three times at 54-h intervals according to the defined regimen (Fig. 1C). Cell cycle analysis of A20 B-cellular lymphoma cells showed that cells accumulated in the G2/M phase on day 7 (the ratio of the minimum number of cells in the S phase to the accumulated maximum number of cells in the G2/M phase) (Fig. 1D). On days 8–9, a mixed cell cycle state is observed, indicating cell release from arrest.

Thus, the analysis of the cell cycle allowed us to determine the time of the final eradication treatment, which leads to the complete destruction of cells. The cytostatic agent is administered at the time point when there is an accumulation of cells in the G2/M phase of the cell cycle, that is, on the 7th day, or 168 h after the first treatment. The DNAmix is administered at the moment of transition from the phase of nucleotide excision repair to the phase of homologous recombination, or in other words, at the moment of maximum accumulation of double-strand breaks, that is, on the 9th day after the start of therapy, or 48 h after the final cytostatic.

As a result of the analysis performed, we established a treatment regimen with CP and DNAmix for A20 B-cellular lymphoma (Fig. 1E).

### Experimental comparison of the effectiveness of tumor treatment using the Karanahan technology and in situ vaccination in the A20 B-cellular lymphoma model

**Figure 2:**
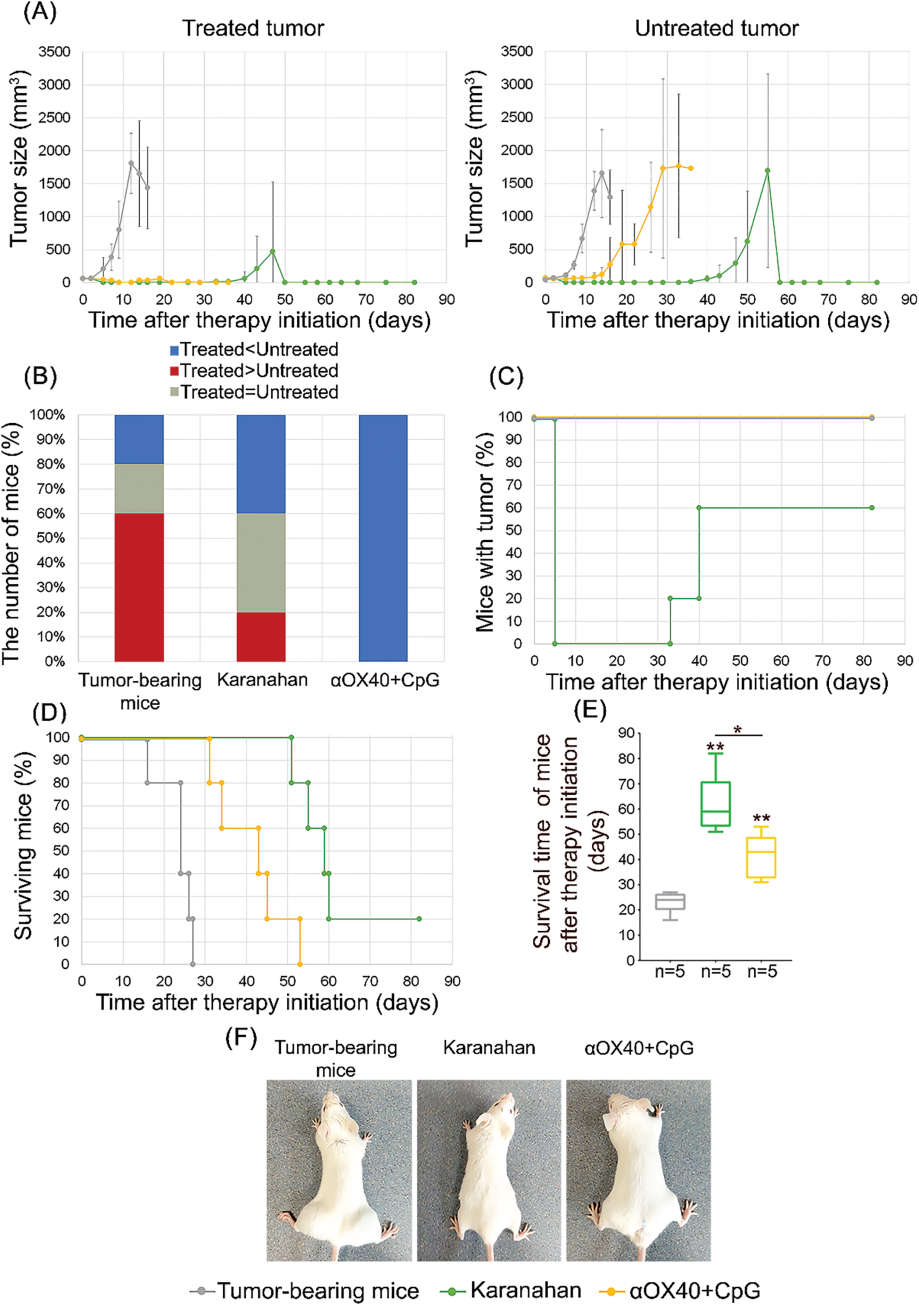
Comparison of the effectiveness of the Karanahan technology and *in situ* vaccination in the model of highly immunogenic A20 B-cellular lymphoma [15]. **(A)** Comparison of tumor growth in experimental groups and tumor-bearing mice (control group), mean values ± standard deviation are presented. **(B)** Percentage of animals at the experiment endpoint: animals with a treated tumor size smaller than that of untreated tumor, animals with the same tumor size on both paws and animals with a larger treated tumor size compared to that of the untreated tumor. **(C)** Changes in the number of mice with a tumor. **(D)** Animal survival. **(E)** Average survival time. * –differences between the groups are significant with *p*-value < 0.05, ** – differences between the groups are significant with *p*-value < 0.01; Mann–Whitney U test. An asterisk without a dash indicates a difference with the control group (tumor-bearing mice). **(F)** Animal appearance.

This section is part of another work [15], which was conducted in parallel with this study; and the obtained data belong to two experimental branches (Fig. 2).

*Treatment of A20 B-cellular lymphoma using the Karanahan technology*. Treatment of A20 B-cellular lymphoma using the Karanahan technology demonstrated complete regression of the developed grafts on day 5 after therapy initiation. On days 33–40, 60% of animals developed relapses, while on average a 5–6-fold delay in growth of both the treated tumor and untreated distant graft was observed compared to the control group (tumor-bearing mice) (Fig. 2A,C,F). By the end of the observation period, i.e., on day 95 after therapy initiation, no tumor in any form was detected in 20% of surviving animals. We noted complete healing from A20 B-cellular lymphoma (Fig. 2С). An analysis of the ratios of the sizes of treated and untreated tumors of experimental and control groups indicates the development of both local innate and adaptive immune responses (Fig. 2A,B).

*Treatment of A20 B-cellular lymphoma using in situ vaccination*. In the group of mice treated using *in situ* vaccination technology, 100% effectiveness was observed only in treated tumors (Fig. 2A). Tumors completely regressed, and no relapses were observed, which indicates activation of the innate immune response. The distant untreated graft after *in situ* vaccination showed a two-fold delay in growth rate compared to the control (tumor-bearing mice) (Fig. 2A,B). No complete regression of untreated tumor site and, therefore, no complete activation of adaptive immunity were observed (Fig. 2B,F). The average survival time of animals treated using *in situ* vaccination technology was almost twice as long as that in the control group (the average survival time of animals was 23 and 41 days in tumor-bearing mice (control) and experimental animals, respectively) (Fig. 2D,E).

Thus, the effectiveness of both approaches in treating experimental A20 B-cellular lymphoma was demonstrated, suggesting the possibility of combining therapies into one therapeutic platform.

**Figure 3:**
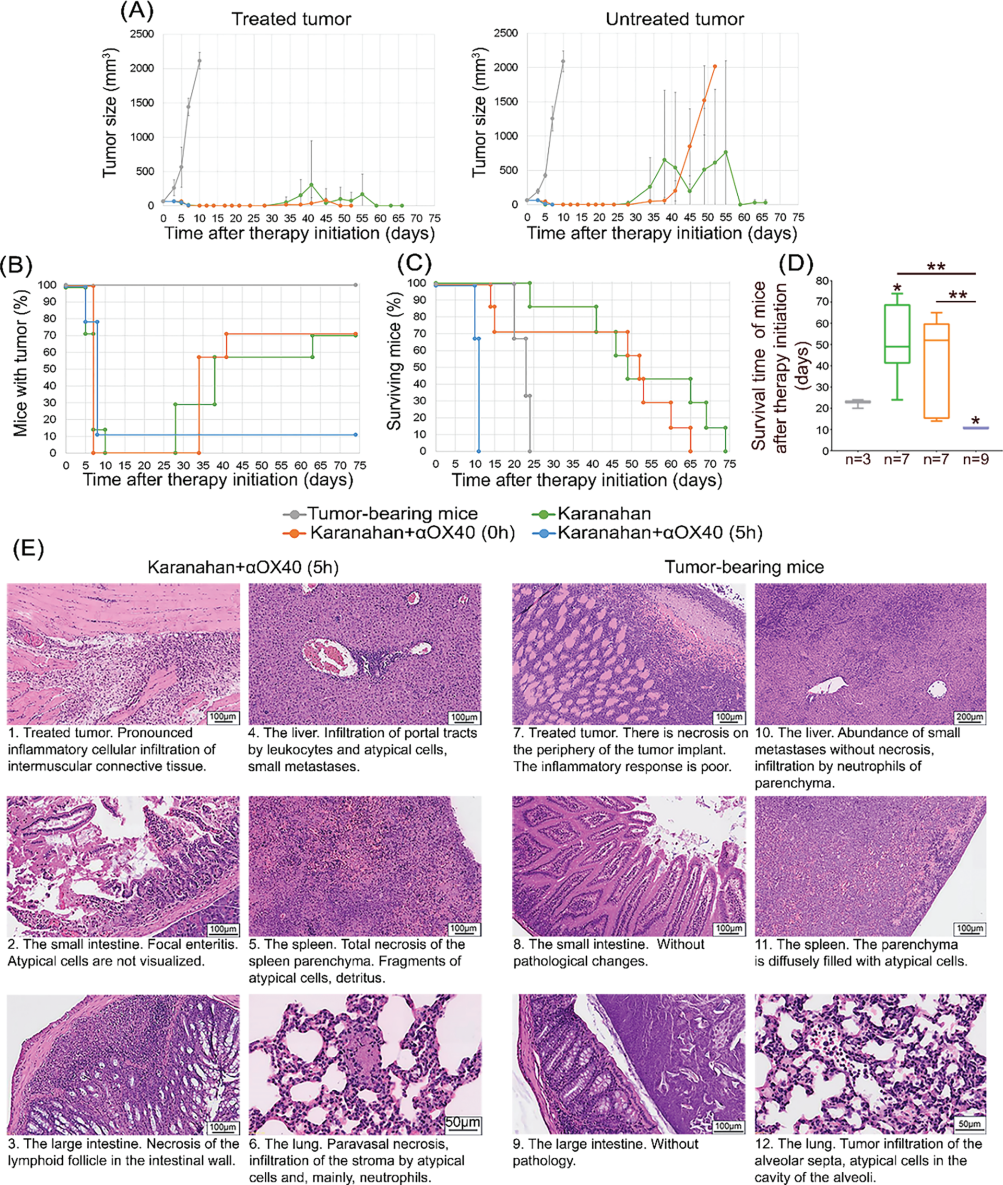
Pilot combination of two technologies for the treatment of A20 B-cellular lymphoma (Karanahan technology and *in situ* vaccination with CpG/αOX40) into one therapeutic regimen with injections of 8 μg αOX40 intratumorally immediately or 5 h after each composite double-stranded DNA preparation (DNAmix) injection. **(A)** Comparison of tumor growth changes in experimental groups and tumor-bearing mice (control); mean values ± standard deviation are presented. **(B)** Changes in the number of mice with a tumor. **(C)** Animal survival. **(D)** Average survival time. * – differences between the groups are significant with *p*-value < 0.05, ** – differences between the groups are significant with *p*-value < 0.01; Mann–Whitney U test. An asterisk without a dash indicates a difference with the control group (tumor-bearing mice). **(E)** Pathomorphological analysis of animal organs. Organs (liver, spleen, small and large intestines, and lungs) and treated tumors were isolated from experimental and control animals (tumor-bearing mice) and fixed in 4% neutral paraformaldehyde. Organ samples were dehydrated in increasing ethanol concentrations, cleared in xylene, and embedded in paraffin. Paraffin sections up to 5 µm thick were stained with hematoxylin and eosin.

We conducted experiments on combining the two technologies (Fig. 3).

### Pilot combination of two modern technologies for tumor treatment: the Karanahan technology and in situ vaccination with CpG/αOX40 in the A20 B-cellular lymphoma model

Treatment of A20 B-cellular lymphoma using the Karanahan technology in the monotherapy mode and in combination with αOX40 administered at the tumor site simultaneously with DNAmix demonstrated complete graft regression on days 7–10 after therapy initiation. On days 28–34, relapses were observed in the above groups. At the endpoint of the experiment, 70% of animals treated using the Karanahan technology in the monotherapy mode and in combination with αOX40 administered at the tumor site simultaneously with DNAmix developed relapses (Fig. 3A,B). A 6-fold delay in the growth of untreated graft in the “Karanahan” group in the monotherapy mode and a 7-fold delay in the growth of untreated graft in the “Karanahan” group with αOX40, introduced into the tumor site simultaneously with DNAmix, were observed compared to the control group (tumor-bearing mice) (Fig. 3A).

The average survival time of animals treated using Karanahan technology in the monotherapy mode and in combination with αOX40 administered at the tumor site simultaneously with DNAmix was 2.4/2 times higher compared to that in tumor-bearing mice, respectively (Fig. 3C,D).

Administration of αOX40 5 h after DNAmix injection resulted in complete graft regression in 90% of the animals by day 8 of the experiment; however, on days 10–11 (in the illustrated experiment), all animals died almost simultaneously (Fig. 3B,C).

We hypothesized that the rapid death of experimental mice after injection of antibodies at the tumor site 5 h after DNAmix administration can be due to immune system hyperactivation and the subsequent development of a systemic inflammatory response.

At the next stage, we conducted numerous analyses aimed at the identification of the causes of accidental death in experimental animals.

*Pathomorphological analysis of animal organs*. To reveal the causes of mouse death, the pilot experiment on combining two modern technologies for tumor therapy was repeated without significant changes, with the exception of shifting the experiment time to the spring. When signs of impending death were noted (hump formation, sudden weight loss, and decreased locomotor activity), mice were sacrificed by cervical dislocation. Organs and tissues were isolated from experimental and control (tumor-bearing mice) animals and fixed in 4% neutral paraformaldehyde for pathomorphological analysis.

The pathomorphological analysis showed the development of a systemic inflammatory process in experimental mice treated using the Karanahan technology combined with αOX40, administered 5 h after DNAmix injection. A pronounced inflammatory cell infiltration of the intramuscular connective tissue is observed at the tumor site. Numerous necrotic foci and abundant neutrophil infiltration are found in the small and large intestine, spleen, and lungs, which indicates the presence of inflammation. Atypical cells are detected in the spleen and lungs.

Necrosis is observed at the tumor site in tumor-bearing mice, but the inflammatory response is weak. No pathological changes were found in the small and large intestine. Atypical cells are noted in the spleen and lungs.

An abundance of small metastases and leukocyte infiltration is observed in the liver of both control (tumor-bearing) and experimental mice (Fig. 3E).

The pathomorphological data obtained indicate the development of inflammatory process throughout the whole body in mice after Karanahan treatment in combination with αOX40, administered 5 h after DNAmix administration. The pattern of pathological changes in analyzed organs and tissues indicates that performed treatments induced a systemic inflammatory response, which always led to multiple organ failure and death.

In the next part of the study, we performed experiments to reveal the causes leading to the development of systemic inflammation and animal death after Karanahan treatment in combination with αOX40, administered 5 h after DNAmix exposure.

### Analysis of blood cell populations in mice treated using the Karanahan technology

*Assessment of the number of immune cells capable of activating a systemic inflammation (CD11b+Ly-6C+, CD11b+Ly-6G+, CD3-NKp46+CD11b+)*. There are three types of cells that, when over-activated and present in large numbers in the peripheral blood, can induce widespread inflammation, detected by pathomorphological analysis of the dead mouse organs. These are macrophages (CD11b+Ly-6C+ monocytes), neutrophils (CD11b+Ly-6G+ granulocytes), and natural killer cells (CD3-NKp46+CD11b+). All these cells possess a powerful arsenal of cytolytic mechanisms, which, when activated, lead to the destruction of specific targets. In the absence of target structures, cytotoxic mononuclear cells can induce lysis of healthy cells and tissue necrosis through unimplemented activated lytic and other mechanisms [18]. In case of an excessive cell release in the blood, this activity results in the formation of multiple necrotic foci, which is observed in dead animals. We assessed the above cell populations in the peripheral blood of mice after Karanahan therapy. Blood was isolated at the “DNAmix+5 h” time point (5 h after each DNAmix injection). After the injections at the selected “DNAmix + 5 h” time points, one animal was selected from the experimental “Karanahan” group (one animal for each time point). Blood isolated from intact and tumor-bearing mice was used as controls. In the first stage, we assessed the number of the above populations.

We found that 5 h after the first and final Karanahan treatments with DNAmix, a significant release of CD11b+Ly-6C+/CD11b+Ly-6G+ myeloid cells (monocytes and granulocytes) into the blood is observed: up to 16% and 8%, respectively. At the same time, no CD11b+Ly-6C+/CD11b+Ly-6G+ cells were found in the blood of intact and tumor-bearing mice. A second, less abundant release (up to 5%) of myeloid cells into the blood is noted 5 h after the second treatment with DNAmix using the Karanahan approach.

**Figure 4:**
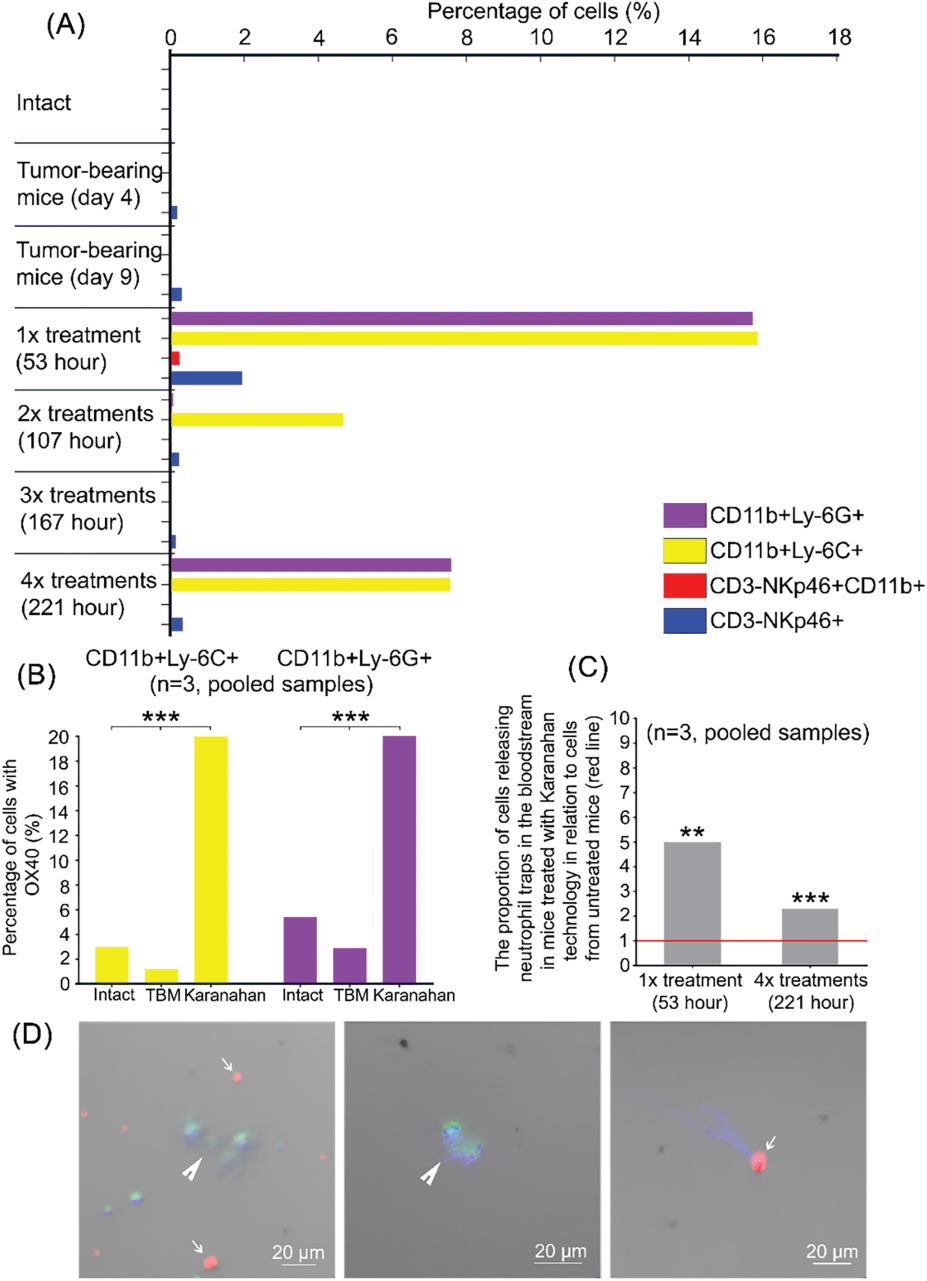
Assessment of changes in the number of CD11b+Ly-6C+/ CD11b+Ly-6G+ and CD3-NKp46+CD11b+/CD3-NKp46+ cells in the blood of experimental animals induced by Karanahan treatment of mice with A20 B-cellular lymphoma. **(A)** Percentage of myeloid-derived cells: CD11b+Ly-6C+ monocytes and CD11b+Ly-6G+ granulocytes, mature CD3-NKp46+CD11b+/immature CD3-NKp46+ natural killer cells in the blood of intact mice and tumor-bearing mice on days 4 and 9 after the experiment initiation and experimental group mice after the 1st, 2nd, 3rd, and the final 4th Karanahan treatments. The animal was sacrificed using the method of cervical dislocation 5 h after composite double-stranded DNA preparation (DNAmix) administration. One animal was used for each time point from the general “Karanahan” group for analysis. **(B)** The percentage of myeloid-derived cells: CD11b+Ly-6C+ monocytes and CD11b+Ly-6G+ granulocytes, carrying OX40 on their surface, on day 6 after the initiation of Karanahan therapy (pooled samples, n = 3). TBM–tumor-bearing mice. *** – differences between the groups are significant with *p*-value < 0.001; analysis of distributions grouped into four-field tables, χ^2^ test. **(C)** Assessment of the number of myeloid-derived cells with the CD11b+ phenotype, bearing the Ly-6G granulocyte marker on their surface, activated to NETosis. The graph presents the proportion of cells releasing neutrophil traps in mice receiving Karanahan treatment in comparison with tumor-bearing mice. Blood was isolated from experimental group mice after the 1st and 4th Karanahan treatments; blood from tumor-bearing mice was isolated in parallel at the same time points. The animal was sacrificed using the method of cervical dislocation for blood analysis 5 h after DNAmix administration. The percentage of CD11b+DAPI+Ly-6G+ cells in the entire CD11b+ cell population was assessed. The figure presents the results of the analysis of pooled blood samples (n = 3). ** – differences between the control (tumor-bearing mice) and experimental groups are significant with *p*-value < 0.01; *** – differences between the control (tumor-bearing mice) and experimental groups are significant with *p*-value < 0.001; analysis of distributions grouped into four-field tables, χ^2^ test. **(D)** Appearance of neutrophil traps. The red signal is CD11b+, the green signal is Ly-6G+; and the blue signal is DAPI (4′,6-diamidino-2-phenylindole). CD11b+ cells are indicated by a thin arrow, and CD11b+DAPI+Ly-6G+ cells are indicated by a thick arrow.

Natural killer cells in the mouse blood isolated 5 h after the first Karanahan treatment with DNAmix consisted of 2% immature cells and 0.2% mature cells. The percentage of immature natural killer cells in the blood of experimental and tumor-bearing mice was at approximately the same level (about 0.25%) (Fig. 4A).

Taken together, our findings indicate that treatment of experimental mice using the Karanahan technology induces activation of the immune system with a robust release of monocytes and granulocytes into the blood. These are the cells that can cause the sudden death of animals upon introduction of αOX40 into the therapy.

*A number of myeloid-derived cells (CD11b+Ly-6C+ monocytes and CD11b+Ly-6G+ granulocytes) carrying FcR and OX40 on their surface*. Immune cells can be activated by specific interaction of an agonist ligand with the corresponding receptor [19,20]. Agonistic antibodies to the OX40 are used in the analyzed system. This fact means that activation of cytolytic mechanisms can be associated with either specific interaction of the antibody epitope OX40 and OX40, the interaction of the Fc fragment of the same antibody and FcR, or as a result of double activation [7,18,21]. In other words, FcR/OX40 interaction on the surface of macrophages and(or) neutrophils with αOX40 can result in activation of the cytolytic activity of these cells and induction of non-specific lysis of the surrounding cells in tissues, which will lead to the formation of numerous necrotic inflammatory foci.

We carried out a series of experiments in order to estimate the number of myeloid immune cells carrying FcR/OX40 on their surface among blood mononuclear cells.

We established that, in the experimental system of the present study (control and experimental mice), 100% of CD11b+Ly-6C+/CD11b+Ly-6G+ cells (which mature into macrophages and neutrophils, respectively) carry FcR on their surface. We showed that Karanahan treatment in mice resulted in a significant increase in the percentage of CD11b+Ly-6C+/CD11b+Ly-6G+ cells carrying OX40 on their surface. For monocytes, this parameter changes as follows: 3% in the blood of intact mice, 1.2% in the blood of tumor-bearing mice, and 20% in the blood of mice receiving Karanahan treatment. For granulocytes, the values are as follows: 5.4% in the blood of intact mice, 2.9% in the blood of tumor-bearing mice, and 21.7% in the blood of mice receiving Karanahan treatment (Fig. 4B).

We can assume that, during the interaction of FcR/OX40 on the surface of macrophages and neutrophils with αOX40, the cytolytic activity of these cells is activated, and non-specific lysis of surrounding cells in the tissues is induced, which leads to the formation of numerous necrotic foci detected by pathomorphological analysis.

OX40L is known to be expressed on the surface of antigen-presenting cells, natural killer cells, activated T-cells, cells of the smooth muscles of the respiratory tract, and vascular endothelial cells. The interaction of FcR on the macrophage and neutrophil surface with the Fc fragment of αOX40 antibodies can lead to the activation of these cells with subsequent interaction of their OX40 with OX40L on the target cell surface. This, in turn, leads to the lysis of target cells (vascular endothelium, smooth muscles of the respiratory tract) and the formation of numerous necrotic foci.

Thus, we showed that FcR and OX40 are present on the surface of monocytes and granulocytes released into the bloodstream at peak values after the Karanahan therapy. A specific interaction with agonistic αOX40 antibodies appears to be a trigger for the development of systemic inflammation in experimental mice.

*Assessment of the number of MDSCs with the CD11b+Ly-6G+ phenotype and activated to NETosis*. One of the mechanisms responsible for the formation of numerous necrotic foci in mice of the experimental group may be the specific activation of neutrophils, which causes their programmed cell death: NETosis with the formation of specific neutrophil trap structures [22–24]. Neutrophil traps promote aggregation of red blood cells in microcapillaries, which causes microvascular thrombosis and, consequently, a systemic inflammatory response.

At this stage, we assessed the percentage of CD11b+DAPI+Ly-6G+ cells in all CD11b+ cells in the blood of experimental and control (tumor-bearing mice) animals. The 1st Karanahan treatment resulted in a 5-fold increase in the number of cells forming neutrophil traps, while the 4th treatment led to a 2.2-fold increase compared to that in tumor-bearing mice (Fig. 4C,D).

We made the following conclusion based on the obtained results. Karanahan treatment of A20 B-cellular lymphoma results in a robust release of CD11b+Ly-6C+ and CD11b+Ly-6G+ cells into the bloodstream at certain time points. Due to the presence of FcR and OX40 on the surface, these cells can be activated and cause either non-specific lysis of nearby cells or lysis of target cells carrying OX40L on their surface. In addition, the Karanahan technology activates NETosis in neutrophils. Apparently, these events are responsible for the formation of widespread necrotic foci and induction of a systemic inflammatory response, leading to multiple organ failure and death of experimental animals.

At the last stage of the study, the therapeutic regimen of antibody administration was adjusted based on the obtained data on peak values for cells with potential cytolytic properties and capable of NETosis in the blood of experimental mice.

### Adjusted combined therapy using the Karanahan technology and in situ vaccination with CpG/αOX40 in the A20 B-cellular lymphoma model

**Figure 5:**
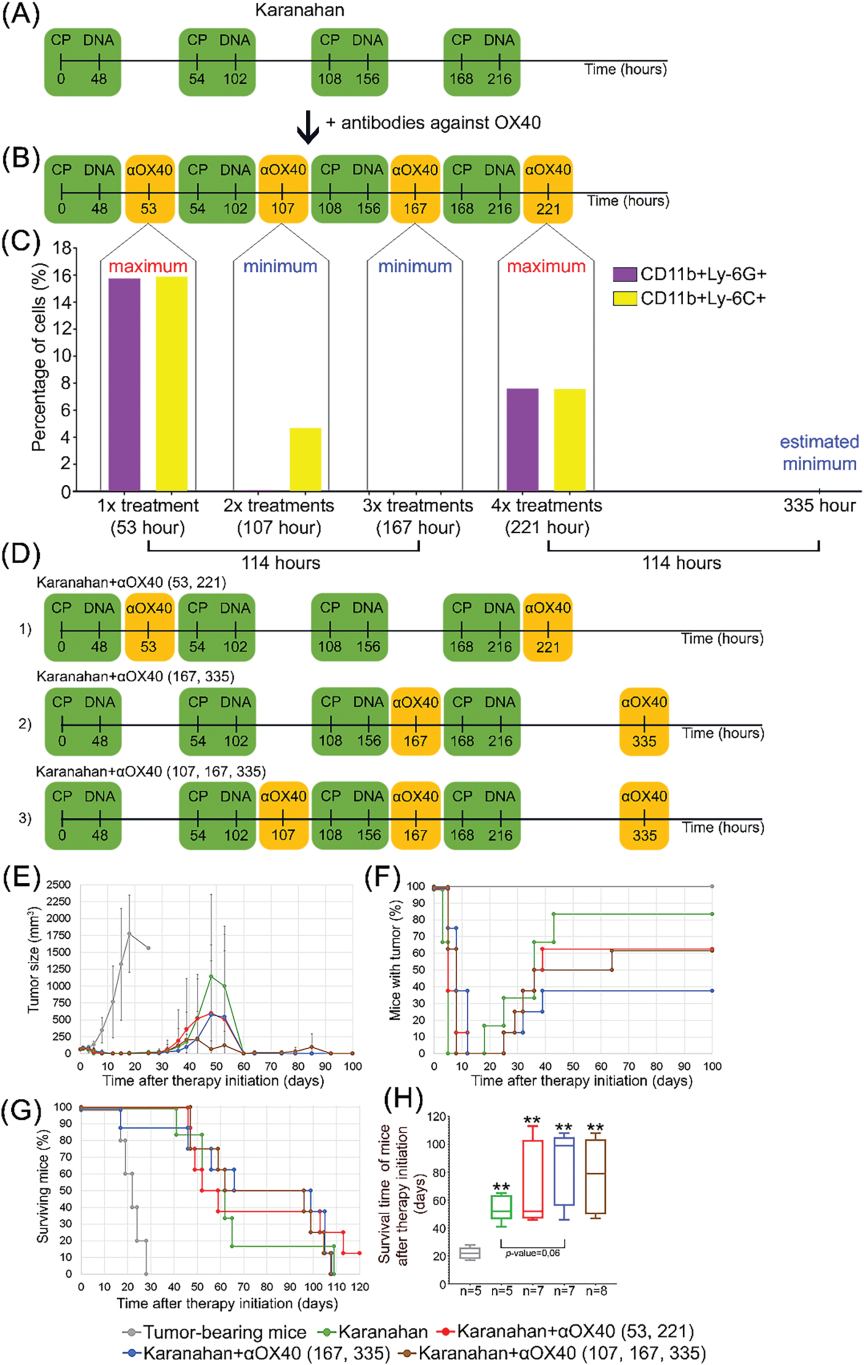
Adjusted combination of two technologies for the treatment of A20 B-cellular lymphoma (Karanahan technology and *in situ* vaccination with CpG/αOX40) into one therapeutic regimen. **(A)** Therapeutic regimen for the treatment of A20 B-cellular lymphoma using the Karanahan technology (in the figure composite double-stranded DNA preparation (DNAmix) is indicated as DNA). **(B)** Therapeutic regimen for the treatment of A20 B-cellular lymphoma using the Karanahan technology in combination with administration of antibodies to the OX40 receptor 5 h after DNAmix injection. **(C)** Percentage of myeloid-derived cells: CD11b+Ly-6C+ monocytes and CD11b+Ly-6G+ granulocytes in the blood isolated from experimental group mice after the 1st, 2nd, 3rd, and the final 4th Karanahan treatments. Two maxima (53 and 221 h time points with a high release of monocytes and granulocytes into the blood) and two minima (107 and 167 h time points with a low release of monocytes and granulocytes into the blood) were identified; a time point with a low release of monocytes and granulocytes into the blood (by analogy with the onset of the minimum release 114 h after the 1st treatment) was also predicted. **(D)** Therapeutic regimens for the treatment of A20 B-cellular lymphoma using the Karanahan technology in combination with *in situ* vaccination with αOX40 administered 5 h after DNA mix injections at certain time points. **(E–H)** Comparison of the effectiveness of several selected therapeutic regimens. **(E)** Comparison of tumor growth changes in experimental groups and tumor-bearing mice, mean values ± standard deviation are presented. **(F)** Changes in the number of mice with a tumor. **(G)** Animal survival. **(H)** Average survival time. ** – differences between the experimental group and the group of tumor-bearing mice are significant with *p*-value < 0.01; Mann–Whitney U test.

Tumor therapy using the Karanahan technology in mouse models includes four procedures (treatments): cytostatic (CP) injection (intraperitoneal) + DNAmix injection (intratumoral) (Fig. 5A). All injections are linked to repair and cell cycles of a specific tumor model.

A significant number of monocytes and granulocytes are released into the blood 5 h after the 1st (53 h) and 4th (221 h) Karanahan treatments (Fig. 5B,C). These cells carry FcR and OX40 on their surface. Interaction of these receptors with αOX40 results in activation of lytic properties of macrophages and neutrophils, leading to target cell lysis. Furthermore, Karanahan treatments activate NETosis in neutrophils. Taken together, these processes induce systemic inflammation and multiple organ failure, leading to animal death. The obtained results were taken into account when developing a new therapeutic regimen for the treatment of A20 B-cellular lymphoma. Injections of αOX40 at the time points of maximum levels of monocytes and granulocytes in the blood of experimental animals were excluded from the new therapeutic regimen.

Two maxima (the aforementioned 53 and 221 h time points with a peak release of monocytes and granulocytes into the blood) and two minima (107 and 167 h time points with a minimum release of monocytes and granulocytes into the blood) were noted. The predicted time point was also selected (335 h, 114 h after the 4th treatment) with a low release of monocytes and granulocytes into the blood (similar to the onset of the minimum release 114 h after the 1st treatment) (Fig. 5C).

Three adjusted experimental regimens were designed to combine the two technologies for the treatment of A20 B-cellular lymphoma (Fig. 5D).

No. 1. Karanahan+αOX40 (53,221) regimen: antibody administration 5 h after the first and fourth treatments (at “two maxima”).

No. 2. Karanahan+αOX40 (167,335) regimen: antibody administration 5 h after the third treatment and at the predicted time point with a low release of monocytes and granulocytes into the blood (at “two minima”).

No. 3. Karanahan+αOX40 (107,167,335) regimen: antibody administration 5 h after the second and third treatments and at the predicted time point with a low release of monocytes and granulocytes into the blood (at “three minima”).

Mouse treatment using the Karanahan monotherapy results in complete tumor regression on day 5 after therapy initiation. Relapses are observed in 83% of mice starting from day 18. The use of the Karanahan technology in combination with αOX40 antibodies administered at “two maxima/two minima” time points in mice results in complete tumor regression on day 12 after therapy initiation. Relapses were noted starting from day 25. Relapses were observed in 62% of mice receiving αOX40 antibodies at “two maxima” and in 37% of mice receiving αOX40 antibodies at “two minima” (Fig. 5E,F). In addition, complete tumor regression was noted on day 8 after therapy initiation in mice receiving Karanahan treatment in combination with αOX40 antibodies administered at “three minima”. A total of 62% of mice developed relapses starting from day 25. According to the results of an analysis in this group of mice, relapses do not develop to large sizes; the average tumor size in this group does not exceed 210 mm^3^. This fact suggests that the cause of mouse death is not tumor development but, apparently, a toxic effect associated with the amount of αOX40 antibodies administered (three injections) (Fig. 5E,F). The average survival time of animals of the three groups receiving Karanahan treatment in combination with αOX40 antibodies is 75 days, which is 3.4 times higher than that of the control group (tumor-bearing mice) and 1.2 times higher than that of the group receiving Karanahan monotherapy (Fig. 5G,H). All three groups exhibited on average a 6.5-fold delay in graft growth compared to the control group (tumor-bearing mice) (Fig. 5E).

Thus, considering the average survival time of animals, the presumed cause of death (either tumor load or antibody dose), and the number of developed relapses, we can conclude that regimen No. 2 is the most effective one. Karanahan+αOX40 (167, 335) is the regimen that involves the administration of αOX40 antibodies 5 h after the third Karanahan treatment and at the predicted time point with a low release of monocytes and granulocytes into the blood (at “two minima”).

We assume that a decrease in the therapy effects in final experiments compared to the first ones is due to the time when the research was conducted: initial experiments were performed during the winter-spring season, while final experiments were conducted in autumn. We believe that differences are associated with seasonal changes in the biological parameters of the experimental system.

## Discussion

The results of this study, which is aimed at assessing the possibility of combining two new technologies into one highly effective anti-cancer therapy, demonstrate that elements of the two selected approaches may conflict with each other and lead to the death of all mice in the corresponding experimental group. The performed analysis indicates that the main cause of this conflict is the interaction of five factors: 1) stimulatory effect of CP; 2) stimulatory effect of DNAmix; 3) subsequent release of a large number of cells with phenotypes CD11b+Ly-6C+ and CD11b+Ly-6G+ into the peripheral blood; 4) administration of αOX40 into the tumor site; 5) time of αOX40 administration. The level of the molecular–cellular conflict was such that all mice in the experimental group died at the same time within 10–11 or 13–14 days after therapy initiation (two repeats, respectively). The pathomorphological analysis showed an induction of a systemic inflammatory response with numerous inflammatory foci in mice. A shift in the time of αOX40 antibody injection to the time point of complete subsidence of the expansion of the peripheral blood by CD11b+Ly-6C+ and CD1b1+Ly-6G+ cells prevents animal death. Furthermore, the new treatment regimen demonstrates the highest synergistic antitumor effect compared to all experimental regimens analyzed in the present study.

Cells with phenotypes CD11b+Ly-6C+ and CD11b+Ly-6G+ belong to MDSCs. MDSCs consist of two large cell groups. These are granulocytic, or polymorphonuclear cells, which are phenotypically and morphologically similar to neutrophils, and monocytic cells, which are phenotypically and morphologically similar to macrophages. In mice, MDSCs are located in the bone marrow, peripheral blood, spleen, liver, lungs, and tumors of various organs [25]. The tumor graft is considered the main source of this group of cells in treated mice. The number of cells in both populations increased to ~16% at the first point of analysis. At the second peak release point, the number of cells in each population was around 8%.

To elucidate the phenomenon, we analyzed, as we believe, all possible, acceptable to the situation, ways of activating cells with the above phenotype that, according to the experiment results, lead to animal death. The sequence of activating events was as follows. Tumor-bearing mice were treated with CP. The treatment induced interstrand crosslink repair. During the demarcation of the two phases of the repair process, mice received a DNAmix. αOX40 were injected 5 h after DNAmix treatment (in the lethal group). The procedures was repeated according to the Karanahan therapy regimen.

According to the results of blood cell population analysis, it is treatment with CP and DNAmix that induces the release of myeloid suppressors into the blood, with the release peak noted 5 h after DNAmix injection. The obtained data do not allow us to determine the main trigger of this event. Both cell types (CD11b+Ly-6C+ and CD11b+Ly-6G+) express two receptors on their surface, namely FcR [26] and OX40 [27]. FcR is present in almost all cells with this phenotype. The number of OX40+ cells increases up to 20% in both cell types and averages 3% in untreated tumor-bearing and intact animals. OX40 is known to reach the cytoplasmic membrane 1–4 h after induction, with a peak release after 48–72 h [28]. We believe that, after CP and DNAmix administration, activation of CD11b+Ly-6C+ and CD11b+Ly-6G+ cells, accompanied by OX40 expression on their cytoplasmic membrane, takes place simultaneously with the release of an excess number of these cells into the bloodstream.

How FcR and OX40 display on the cell surface can affect the activity of the two cell populations.

Cells with the CD11b+Ly-6C+ phenotype belong to the monocyte fraction, they mature into macrophages [29]. The maturity of CD11b+Ly-6C+ MDSCs has not been functionally determined. However, the expression of FcR and OX40 receptors may indicate their mature state. FcR is responsible for the macrophage phagocytic activity [30]. The presence of OX40 on the surface of this cell type has not previously been shown. It is possible that this stimulatory effect is due to the CP action. The engagement with αOX40 both in the FcR/Fc fragment and OX40/αOX40 variants can be accompanied by activation of macrophage cytolytic activity in the form of production of NO, free radicals, and pro-inflammatory cytokines [31]. Furthermore, peak releases of these cells into the blood may induce non-specific lysis of any nearby cells, eventually resulting in the formation of numerous necrotic foci. Macrophages can be also activated by the engagement with antibodies through FcR with simultaneous interaction of phagocytes with OX40L+ cells. As mentioned above, OX40L is expressed on the surface of antigen-presenting cells, natural killer cells, activated T-cells, cells of the smooth muscles of the respiratory tract, and vascular endothelial cells [32]. This double interaction is characterized by both active phagocytosis and targeted destruction of the target cell (directed cytoreducing action of the macrophage). Destruction of vascular endothelium and smooth respiratory muscles seems to be critical for organism survival. In this scenario, numerous necrotic foci are also formed, and inflammation progresses.

Cells with the CD11b+Ly-6G+ phenotype belong to polymorphonuclear leukocytes, which mature into neutrophils [33]. Neutrophils are one of the most aggressive cell types in the immune system; they can lyse any pathogens and cells of their own body when activated. The killing (lytic) potential of neutrophils is presented by a broad arsenal of molecular and cellular mechanisms, including secretion of protein factors, production of hypochloric acid and reactive oxygen species, antibody-dependent cellular cytotoxicity, release of cytokines TNFα, INFγ, IL-1b, 2, 4, and 12, which enhance the cytolytic activity of neutrophils, and formation of neutrophil traps (NETosis) [34–36].

A mature neutrophil has the FcR receptor on its surface, which is responsible for the mechanism of antibody-dependent cellular cytotoxicity. In the ideal scenario, antibody-dependent cellular cytotoxicity takes place when the neutrophil Fc receptor binds to antibodies that have formed immune complexes with antigens on the cell surface. This binding leads to synapse formation and degranulation, resulting in cell death [37].

There is a study showing the presence of OX40 in neutrophils [38]. The engagement of neutrophil OX40 with OX40L is accompanied by an increase in overall neutrophil survival due to a delay in physiological apoptosis.

As mentioned above, stimulation with CP and DNAmix results in a sharp increase in the number of peripheral blood granulocytes 5 h after DNAmix administration. The injection of αOX40 at this time point leads to the synchronous death of mice due to a systemic inflammatory response. Activated by the treatment with CP and DNAmix, neutrophils carrying both FcR and OX40 can induce non-specific lysis of OX40L-expressing cells in the presence of antibodies; these OX40L-expressing cells include antigen-presenting cells, natural killer cells, activated T-cells, cells of the smooth muscles of the respiratory tract, and vascular endothelial cells [32]. Having bound to the neutrophil, antibodies activate the degranulation mechanism, and the engagement with OX40L determines the target cell the lytic synapse is formed with.

Thus, two types of phagocytes destroy OX40L+ cells in unison. Of these OX40L+ cells, vascular endothelial cells, which form the body’s transport system, and cells of the smooth muscles of the respiratory tract, which regulate the oxygen supply to the body, are the most vulnerable ones. As evidenced by animal experiments, this type of damage is incompatible with life.

The results indicate that around 30% to 55% of neutrophils isolated at the indicated experimental point can undergo active NETosis [39]. This fact additionally supports the hypothesis on induction of the systemic inflammatory response identified during pathomorphological analysis by aggressive phagocytes released into the bloodstream in large numbers as a result of the Karanahan therapy. αOX40 served as the trigger for neutrophil trap formation.

Identification of time points of the maximum presence and a drop in the number of phagocytes in the blood made it possible to adjust the antibody administration. The experiment confirmed the above hypothesis that αOX40 administration at peak values of the parameter (number of phagocytes in the peripheral blood) is more toxic compared to that of other time regimens. The insufficiently pronounced mouse death in the most destructive regimen is apparently due to the following circumstances. This particular experiment did not assess the presence of phagocytes in the peripheral blood. Instead, data from previous experiments were used as the basis. The experiment was conducted in the late autumn–early winter period; we believe that the time of phagocyte release to the periphery was shifted as a result of seasonal cycle variability, which we demonstrated in our previous studies [12].

There are known and characterized seasonal and circadian variations in biological systems [40–43]. Seasonal variations in microRNA gene expression [44,45] and TNF production [46] have been described. Seasonal correlations in the incidence of primary cancers, which are associated with the level of vitamin D produced [47,48], and postoperative pulmonary metastases in breast cancer [49] have been reported. Many biochemical parameters (serum melatonin and steroid receptor levels) of breast cancer are known to be seasonal [50]. There are also many experimental studies characterizing seasonal and circadian variations in the proliferative activity of various cell types, including stem cells [51,52]. Such fluctuations, as is known, can be associated with seasonal changes in the release of cortisol by the hypothalamic–pituitary–adrenal axis [53–57].

Our early experiments demonstrated the seasonal activity of the synergistic effect of CP and a native human DNA preparation on hematopoietic stem cells. The lymphoid germ of hematopoiesis is destroyed in winter due to a synergistic effect, and the so-called “death window” interval starts, which ends in summer [12]. Experiments in the Krebs-2 model showed that the repair cycle of tumor cells changes depending on the season and that the Karanahan therapy’s effectiveness depends on this change. Experiments in the Lewis carcinoma model also revealed the effects of seasonal and annual variability of Karanahan therapy effectiveness. Pronounced changes in the effectiveness of tumor reduction, the incidence of relapses, and their sustainable development were observed in experiments with two transplanted and one treated grafts conducted in the spring and autumn of the same year [12].

The correction of any chromatin damage directly depends on the process of cell division. Seasonal and annual changes in hematopoietic stem cells, Krebs-2 carcinoma, Lewis carcinoma, and the model A20 B-cellular lymphoma/ BALB/c mouse system are assumed to be associated with fluctuations in the duration of the proliferative cycle of cells. This, in turn, leads to significant changes in main Karanahan technology platforms and a decrease in their effectiveness [12].

However, the toxic effect of the new therapeutic concept was also observed in the final series of experiments, which we suppose were conducted at a non-optimal time for therapy. Administration of antibodies at time points with minimal blood levels of phagocytes, established based on previous measurements, increased the average survival time in mice and significantly (up to 63%) increased the percentage of tumor-free animals compared to other groups.

### The proposed integral mechanism of synergistic anticancer action of two modern approaches

We believe that the mechanism of the synergistic antitumor effect is guided by several independent vectors forming an integrated therapeutic impact in the tumor nidus, resulting in tumor regression. Due to the combined effect of CP and DNAmix, CSCs are eradicated from the tumor. Along with being involved in the eradication of CSCs, the cytostatic agent induces the degradation of committed cells in the tumor nidus. An enormous amount of tumor antigens appear in the tumor-associated stroma. Simultaneously, due to intratumoral injections, DNAmix activates dendritic cells of the tumor-associated stroma identically to CpG [7]; having internalized tumor antigens, these dendritic cells migrate to lymph nodes. At the same time, anti-OX40 antibodies interact with the OX40 receptor on the surface of T cells, thus enhancing the lifetime of future killer cells. Having met antigen-presenting dendritic cells and received the signal, mature cytotoxic lymphocytes lyse tumor cells in the tumor growth areas.

## Conclusion

Our findings confirm the fundamental, experimentally demonstrated feasibility of combining the Karanahan technology and *in situ* vaccination with CpG/αOX40. The main condition for the safety and effectiveness of this combination is continuous (every six hours) monitoring of the number of phagocytes in the peripheral blood and determination of the minimum values after peak releases for timely antibody injection. Apparently, the main killing vectors of both approaches determine the positive synergistic effect of the two therapies. For future clinical application of the proposed approach, it is important that one can make a justified choice of either the monotherapy or the synergistic treatment mode. In the case of immunogenic tumors, the choice can be to combine several techniques and activate adaptive immunity. For non-immunogenic tumors, it would be more adequate to choose either the Karanahan technology in the monotherapy mode or combine it with factors activating innate immunity.

## Data Availability

All data generated or analyzed during this study are included in this published article.

## Abbreviations

CSC: Cancer stem cells
DNAmix: Composite double-stranded DNA preparation
CP: Cyclophosphamide
TAMRA: Tetramethylrhodamine
dsDNA: Double-stranded DNA
MMC: Mitomycin C
FcR: Fc receptor
MDSC: Myeloid-derived suppressor cell
CpG: CG-rich oligodeoxynucleotide
TNFRSF: Tumor necrosis factor receptor superfamily
